# Supplementary material for: Essential metabolism for a minimal cell
Source: eLife. 2019 Jan 18;8:e36842. doi: 10.7554/eLife.36842 (PMC6609329; doi:10.7554/eLife.36842)
Supplement: Supplementary file 1. [file elife-36842-supp1.docx]

**SUPPLEMENTARY FILE 1A** Hierarchy used in gene function classification. Adapted from Liebermeister et al. [1] for mycoplasmas.

| Primary | Secondary | Tertiary |  |
| --- | --- | --- | --- |
| Genetic Information Processing | Transcription | Transcription machinery |  |
|  |  | Transcription factors |  |
|  | Translation | Ribosome |  |
|  |  | Translation factors |  |
|  |  | tRNA biogenesis |  |
|  |  | tRNA loading |  |
|  |  | Ribosome biogenesis |  |
|  | Folding, Sorting and Degradation | Protease inhibitors |  |
|  |  | Chaperones and folding catalysts |  |
|  |  | Protein export |  |
|  |  | Peptidases |  |
|  |  | RNA degradation |  |
|  | DNA Maintenance | Viral proteins |  |
|  |  | DNA replication |  |
|  |  | DNA replication complex |  |
|  |  | DNA replication control |  |
|  |  | Chromosome-related |  |
|  |  | Base excision repair |  |
|  |  | Nucleotide excision repair |  |
|  |  | Mismatch repair |  |
|  |  | Homologous recombination |  |
|  |  | DNA repair and recombination proteins |  |
| Environmental Information Processing | Signal Transduction | Stress response |  |
|  |  | Quorum sensing |  |
|  |  | Other signaling pathways |  |
|  |  | Two-component system |  |
| Cellular Processes | Cytoskeleton | Cytoskeleton proteins |  |
|  | Cell Motility | Bacterial chemotaxis |  |
|  |  | Bacterial motility proteins |  |
|  | Cell Growth | Biofilm formation |  |
|  |  | Cell division |  |
|  | Defense | Prokaryotic defense system |  |
| Metabolism | Membrane Transport | Inorganic |  |
|  |  | Cofactor |  |
|  |  | Amino acid and peptides |  |
|  |  | Nucleic acids |  |
|  |  | Carbohydrates |  |
|  |  | Lipids |  |
|  |  | Polyamines |  |
|  |  | Drug resistance |  |
|  |  | Bacteriocin |  |
|  |  | Other Transport |  |
|  |  | Generic |  |
|  |  | Secretion system |  |
|  | Central Carbon Metabolism | Glycolysis |  |
|  |  | TCA cycle and anaplerotic enzymes |  |
|  |  | Pentose phosphate metabolism |  |
|  |  | Carbohydrate metabolism |  |
|  |  | Nucleotide metabolism |  |
|  |  | Pyruvate metabolism |  |
|  |  | Butanoate metabolism |  |
|  |  | Other central metabolism enzymes |  |
|  | Energy Metabolism | Oxidative phosphorylation |  |
|  |  | Methane metabolism |  |
|  |  | Sulfur metabolism |  |
|  |  | Other |  |
|  | Biosynthesis | Lipid metabolism |  |
|  |  | Amino acid metabolism |  |

**SUPPLEMENTARY FILE 1A** (cont.)

| Primary | Secondary | Tertiary |  |
| --- | --- | --- | --- |
|  |  | Glycan metabolism Cofactor biosynthesis  Biosynthesis of other secondary metabolites |  |
|  | Other Enzymes | Other enzymes Protein kinases |  |
| Human Diseases | Drug resistance | Antimicrobial resistance genes |  |
|  | Infectious Diseases | Virulence factors  Bacterial invasion of epithelial cells Bacterial toxins |  |
| Unclear | Kegg ortholog defined | Function unknown  General function prediction only |  |
|  | No Kegg ortholog | Function unknown |  |

**SUPPLEMENTARY FILE 1B** Breakdown of the JCVI-syn3A metabolic reconstruction. Reactions in subsystem “Exchange” act as sources (sinks) for metabolites taken up from (secreted into) the medium. The other subsystems are discussed in detail in the main text, Section 2.2 and Section 2.3.

| Subsystem | Reactions | Genes |
| --- | --- | --- |
| Transport | 86 | 46 |
| Amino acid metabolism | 43 | 29 |
| Central metabolism | 32 | 26 |
| Nucleotide metabolism | 50 | 21 |
| Lipid metabolism | 19 | 20 |
| Cofactor metabolism | 14 | 10 |
| Macromolecules | 8 | 3 |
| Exchange | 85 | 0 |
| Biomass production | 1 | 0 |

**SUPPLEMENTARY FILE 1C** Breakdown of protein coding genes in *M. pneumoniae* [2] into functional classes.

|  | | | Protei | n |  | Genes | |  |  |
| --- | --- | --- | --- | --- | --- | --- | --- | --- | --- |
|  | Functional | hierarchy | % # | unique |  | % # | unique | # model |  |
|  | Cellular Processes | Cell Growth | 0.28 | 2 |  | 0.29 | 2 | 0 |  |
|  |  | Cell Motility | 0.23 | 1 |  | 0.15 | 1 | 0 |  |
|  |  | Defense | 0.56 | 1 |  | 0.15 | 1 | 0 |  |
|  |  | *Subtotal* | 1.06 | 4 |  | 0.58 | 4 | 0 |  |
|  | Genetic Information Processing | DNA Maintenance | 4.25 | 31 |  | 5.96 | 41 | 5 |  |
|  |  | Folding, Sorting and Degradation | 12.65 | 27 |  | 4.36 | 30 | 5 |  |
|  |  | Transcription | 4.29 | 13 |  | 1.89 | 13 | 1 |  |
|  |  | Translation | 26.44 | 99 |  | 17.59 | 121 | 25 |  |
|  |  | *Subtotal* | 47.62 | 170 |  | 29.80 | 205 | 36 |  |
|  | Human Diseases | Infectious Diseases | 3.41 | 7 |  | 1.02 | 7 | 0 |  |
|  | Metabolism | Biosynthesis | 3.35 | 23 |  | 4.22 | 29 | 21 |  |
|  |  | Central Carbon Metabolism | 22.77 | 49 |  | 7.70 | 53 | 51 |  |
|  |  | Energy Metabolism | 0.01 | 1 |  | 0.15 | 1 | 0 |  |
|  |  | Membrane Transport | 4.70 | 41 |  | 9.01 | 62 | 25 |  |
|  |  | Other Enzymes | 3.75 | 9 |  | 2.33 | 16 | 3 |  |
|  |  | *Subtotal* | 34.59 | 123 |  | 23.40 | 161 | 100 |  |
|  | Unclear | Kegg ortholog defined | 0.24 | 5 |  | 1.31 | 9 | 1 |  |
|  |  | No Kegg ortholog | 13.08 | 104 |  | 43.90 | 302 | 8 |  |
|  |  | *Subtotal* | 13.32 | 109 |  | 45.20 | 311 | 9 |  |
|  | *Total* |  | 100.00 | 413 |  | 100.00 | 688 | 145 |  |

**SUPPLEMENTARY FILE 1D** Breakdown of protein coding genes in *E. coli* [3] into functional classes.

|  |  | Protei | n |  | Genes | |  |
| --- | --- | --- | --- | --- | --- | --- | --- |
|  | Functional hierarchy | % # | unique |  | % # | unique |  |
|  | Cellular Processes Cell Growth | 1.09 | 41 |  | 0.88 | 41 |  |
|  | Cell Motility | 1.07 | 39 |  | 0.84 | 39 |  |
|  | Cytoskeleton | 0.90 | 32 |  | 0.69 | 32 |  |
|  | Defense | 0.45 | 34 |  | 0.75 | 35 |  |
|  | *Subtotal* | 3.52 | 146 |  | 3.17 | 147 |  |
|  | Environmental Information Processing Signal Transduction | 2.58 | 134 |  | 2.89 | 134 |  |
|  | Genetic Information Processing DNA Maintenance | 4.47 | 187 |  | 4.03 | 187 |  |
|  | Folding, Sorting and Degradation | 6.65 | 184 |  | 3.97 | 184 |  |
|  | Transcription | 4.69 | 191 |  | 4.14 | 192 |  |
|  | Translation | 9.99 | 229 |  | 4.94 | 229 |  |
|  | *Subtotal* | 25.80 | 791 |  | 17.08 | 792 |  |
|  | Human Diseases Drug resistance | 0.25 | 14 |  | 0.30 | 14 |  |
|  | Infectious Diseases | 0.04 | 3 |  | 0.06 | 3 |  |
|  | *Subtotal* | 0.29 | 17 |  | 0.37 | 17 |  |
|  | Metabolism Biosynthesis | 17.28 | 528 |  | 11.39 | 528 |  |
|  | Central Carbon Metabolism | 11.80 | 372 |  | 8.02 | 372 |  |
|  | Energy Metabolism | 2.26 | 84 |  | 1.81 | 84 |  |
|  | Membrane Transport | 11.44 | 594 |  | 12.81 | 594 |  |
|  | Other Enzymes | 4.49 | 189 |  | 4.08 | 189 |  |
|  | *Subtotal* | 47.27 | 1767 |  | 38.11 | 1767 |  |
|  | Unclear Kegg ortholog defined | 3.89 | 180 |  | 3.88 | 180 |  |
|  | No Kegg ortholog | 16.66 | 1161 |  | 34.51 | 1600 |  |
|  | *Subtotal* | 20.54 | 1341 |  | 38.39 | 1780 |  |
|  | *Total* | 100.00 | 4196 |  | 100.00 | 4637 |  |

**References**

1. Liebermeister W, Noor E, Flamholz A, Davidi D, Bernhardt J, and Milo R (2014). Visual account of protein investment in cellular functions. *Proceedings of the National Academy of Sciences*, **111**(23), pp. 8488–8493. doi:[10.1073/pnas.1314810111](http://dx.doi.org/10.1073/pnas.1314810111).
2. Himmelreich R, Hilbert H, Plagens H, Pirkl E, Li B, Herrmann R, Dandekar T, Huynen M, Regula J, Ueberle B, Zimmermann C, Andrade M, Doerks T, Sanchez-Pulido L, Snel B, Suyama M, Yuan Y, Herrmann R, and Bork P (2014). Mycoplasma pneumoniae M129, complete genome. URL [www.ncbi.nlm.nih.gov/nuccore/U00089.2](http://www.ncbi.nlm.nih.gov/nuccore/U00089.2). Accession no. U00089.2.
3. Jeong H, Barbe V, Vallenet D, Choi SH, Lee C, Lee SW, Vacherie B, Yoon S, Yu DS, Cattolico L, Hur CG, Park HS, Segurens B, Blot M, Schneider D, Studier F, Oh T, Lenski R, Daegelen P, and Kim J (2017). Escherichia coli B str. REL606, complete genome. URL [www.ncbi.nlm.nih.gov/nuccore/NC_012967.1](http://www.ncbi.nlm.nih.gov/nuccore/NC_012967.1). Accession no. NC 012967.1.
