# Supplementary material for: Essential metabolism for a minimal cell
Source: eLife. 2019 Jan 18;8:e36842. doi: 10.7554/eLife.36842 (PMC6609329; doi:10.7554/eLife.36842)
Supplement: Transparent reporting form [file elife-36842-transrepform.pdf]

eLIFE
1st Floor
24 Hills Road
Cambridge CB2 1JP, UK
P 01223 855340
W elifesciences.org
T @elife

eLife's transparent reporting form

We encourage authors to provide detailed information *within their submission* to facilitate the interpretation and replication of experiments. Authors can upload supporting documentation to indicate the use of appropriate reporting guidelines for health-related research (see EQUATOR Network), life science research (see the BioSharing Information Resource), or the ARRIVE guidelines for reporting work involving animal research. Where applicable, authors should refer to any relevant reporting standards documents in this form.

If you have any questions, please consult our Journal Policies and/or contact us: editorial@elifesciences.org.

Sample-size estimation

- You should state whether an appropriate sample size was computed when the study was being designed
- You should state the statistical method of sample size computation and any required assumptions
- If no explicit power analysis was used, you should describe how you decided what sample (replicate) size (number) to use

Please outline where this information can be found within the submission (e.g., sections or figure legends), or explain why this information doesn't apply to your submission:
Proteomics: No explicit power analysis was used. Three biological replicates were analysed for each time point along the growth curve. This is also stated in the proteomics Methods section (p. 44).
Transposon mutagenesis: A single experiment was performed. As this yielded ~92,000 insertion events across a genome with 493 genes, it was deemed to yield sufficient statistics. This is also stated in the transposon mutagenesis Methods section (p. 42).
Growth curve measurements: A single experiment comparing JCVI-syn1.0 and JCVI-syn3A under identical conditions was performed. The accuracy and reproducibility of the measurements (reflected in the R^2 values reported in Fig. S5) allowed the use of single samples, as also observed previously (Hutchison *et al.*, Ref. 12). This is also stated in the growth curve Methods section (p. 42).

Replicates

- You should report how often each experiment was performed
- You should include a definition of biological versus technical replication
- The data obtained should be provided and sufficient information should be provided to indicate the number of independent biological and/or technical replicates
- If you encountered any outliers, you should describe how these were handled
- Criteria for exclusion/inclusion of data should be clearly stated
- High-throughput sequence data should be uploaded before submission, with a private link for reviewers provided (these are available from both GEO and ArrayExpress)

Please outline where this information can be found within the submission (e.g., sections or figure legends), or explain why this information doesn't apply to your submission:
Proteomics: Three biological replicates were analysed per time point. Here, 'biological replicates' means that the samples were derived from independently grown cultures. The criteria for data filtering are described in the proteomics Methods section on p. 45.

eLife Sciences Publications, Ltd is a limited liability non-profit non-stock corporation incorporated in the State of Delaware, USA, with company number 5030732, and is registered in the UK with company number FC030576 and branch number BR015634 at the address 1st Floor, 24 Hills Road, Cambridge CB2 1JP | August 2014

Spreadsheet S4 contains data for all time points and biological replicates. Data were uploaded to MassIVE (massive.ucsd.edu) with dataset identifier MSV000081687 and ProteomeXchange with dataset identifier PXD008159.
Transposon mutagenesis: A single experiment was performed, as discussed in the transposon mutagenesis Methods section (p. 42). Insertions in sequences repeated in the genome were excluded from all further analyses, since they could not be unequivocally assigned to a single gene. This is also clarified in the Methods section on p. 43. All transposon insertion sites are listed in Spreadsheet S1 and the total numbers of insertions per gene in each passage are contained in Spreadsheet S2.
Growth curve measurements: A single experiment comparing JCVI-syn1.0 and JCVI-syn3A under identical conditions was performed; the resulting curves are shown in Fig. S5.

Statistical reporting

- Statistical analysis methods should be described and justified
- Raw data should be presented in figures whenever informative to do so (typically when N per group is less than 10)
- For each experiment, you should identify the statistical tests used, exact values of N, definitions of center, methods of multiple test correction, and dispersion and precision measures (e.g., mean, median, SD, SEM, confidence intervals; and, for the major substantive results, a measure of effect size (e.g., Pearson's r, Cohen's d)
- Report exact p-values wherever possible alongside the summary statistics and 95% confidence intervals. These should be reported for all key questions and not only when the p-value is less than 0.05.

Please outline where this information can be found within the submission (e.g., sections or figure legends), or explain why this information doesn't apply to your submission:
The statistical analysis of the transposon mutagenesis data is described in the Methods section on Tn5 mutagenesis, subsection "Classification of genes" (p. 43).

(For large datasets, or papers with a very large number of statistical tests, you may upload a single table file with tests, Ns, etc., with reference to sections in the manuscript.)

Group allocation

- Indicate how samples were allocated into experimental groups (in the case of clinical studies, please specify allocation to treatment method); if randomization was used, please also state if restricted randomization was applied
- Indicate if masking was used during group allocation, data collection and/or data analysis

Please outline where this information can be found within the submission (e.g., sections or figure legends), or explain why this information doesn't apply to your submission:
N/A

Additional data files ("source data")

- We encourage you to upload relevant additional data files, such as numerical data that are represented as a graph in a figure, or as a summary table
- Where provided, these should be in the most useful format, and they can be uploaded as "Source data" files linked to a main figure or table
- Include model definition files including the full list of parameters used
- Include code used for data analysis (e.g., R, MatLab)
- Avoid stating that data files are "available upon request"

Please indicate the figures or tables for which source data files have been provided:
